# 

**Published:** 1908-06-06

**Authors:** 


### The Whisky Commission.

After a long series of sittings, extending over many weeks, during which the evidence has been almost entirely that of representatives of the trade, the Royal Commission on Whisky has begun to take further evidence from members of the medical profession as to the physiological and pathological effects of different kinds of spirits. Sir William Church and Dr. Ralph Stockman each gave evidence on June 2. Dr. Stockman stated that he had made experiments on rabbits with pot still whisky, patent still whisky, and pure diluted alcohol, which demonstrated to him satisfactorily that none of these substances exercised any appreciable effect on the heart and circulation if given in reasonable doses. He concluded that any further observations to be satisfactory must be carried out on men. For this purpose three young medical men, practically abstainers, constituted themselves the subjects of experiment. The observations were made with—(1) a highly flavoured Highland malt pot still whisky, eight years old; (2) patent still whisky, two years old; (3) patent still whisky, eight years old; and (4) pure alcohol diluted with water. An account of the observations was published in the *British Medical Journal* of May 18, 1907, by Drs. Charteris and Cathcart. After these observations were published the witness became anxious to test the matter more thoroughly, especially in regard to the actions of distillates and residues of pot still whisky and brandy. He himself became the subject of experiment. The alcoholic substances used were—(1) a highly flavoured Highland malt pot still whisky, nine years old, matured in plain wood, containing 63 per cent. alcohol by volume; (2) a patent still whisky, three years old, containing 63 per cent. alcohol by volume; (3) brandy, twelve years old, guaranteed to contain "the ethers," and containing 46 per cent. alcohol by volume; (4) absolute alcohol, diluted with water to 63 per cent. alcohol by volume; and (5) distillates and residues from these (1, 2, and 3). Of (1), (2), and (4) 2 oz. were swallowed at one draught, diluted with 2 oz. of water; and of (3) $2\frac{3}{4}$ oz. were diluted with water to 4 oz. and swallowed. This equalled in each case 1.2 oz. by volume of absolute alcohol. The experiments seemed to show that with pot still whisky, patent still whisky, brandy, and diluted pure alcohol—(1) the action is essentially that of alcohol; (2) the action on the pulse-rate, blood pressure, and nervous system, is not appreciably influenced by the higher alcohols, ether, and other similar bodies present; (3) when taken under conditions of perfect quietude, they do not influence the pulse-rate or blood pressure, the action seems to be essentially a depressant one on the brain; and (4) the initial flushing is an action common to all these substances and many other bodies, and is reflex in character.

### THE GRADUATES' MEDICAL SCHOOLS.

DIARY FOR WEEK JUNE 8 TO 13.

**THE POST-GRADUATE COLLEGE, West London Hospital, Hammersmith, W.**

**At 12.15 p.m.**

**June 12,** Dr. Pritchard, **Practical Medicine.**

**At 5 p.m.**

**June 9,** Dr. Pritchard, **The Diagnosis and Treatment of Arteriosclerosis.**

**June 10,** Dr. Beddard, **Medicine, III.**

**June 11,** Mr. Baldwin, **Practical Surgery, III.**

**June 12,** Dr. Abraham, **Cases of Skin Disease.**

**MEDICAL GRADUATES' COLLEGE AND POLYCLINIC, Chenies Street, W.C.**

**At 5.15 p.m.**

**June 9,** Dr. Jas. Collier, **The Treatment of Neuritis.**

**June 10,** Dr. French, **Points in connection with Intercurrent Medical Illnesses during Pregnancy.**

**June 11,** Prof. Hewlett, **The Diagnosis of Syphilis by the Antigen Method.**

**LONDON SCHOOL OF CLINICAL MEDICINE, Seamen's Hospital, Greenwich, S.E.**

**At 2.15 p.m.**

**June 10,** Dr. F. Taylor, **Jaundice.**

**At 3.15 p.m.**

**June 12,** Mr. McGavin, **The Radical Cure of Complete Procidentia Uteri.**

**NORTH-EAST LONDON POST-GRADUATE COLLEGE, Prince of Wales's General Hospital, Tottenham, N.**

**At 4.30 p.m.**

**June 11,** Dr. Giles, **Demonstration of Gynæcological Operations.**

**THE HOSPITAL FOR SICK CHILDREN, Great Ormond Street, W.C.**

**At 4 p.m.**

**June 11,** Mr. Parsons, **Conjunctivitis in Children.**

**MOUNT VERNON HOSPITAL, Fitzroy Square.**

**At 5 p.m.**

**June 11,** Dr. Whiting, **Arrested Tuberculosis of the Lungs after Sanatorium and other Treatment.**

**CHARING CROSS HOSPITAL, W.C.**

Post-Graduate Demonstrations.

**At 4 p.m.**

**June 11,** Dr. I. Bruce, **Electrical.**

**THE THROAT HOSPITAL, Golden Square, W.**

**At 5.30 p.m.,**

**June 11,** Mr. Rose, **Treatment of Chronic Suppuration of the Accessory Sinuses of the Nose.**

**NATIONAL HOSPITAL FOR THE PARALYSED AND EPILEPTIC, Queen Square, Bloomsbury.**

**At 3.30 p.m.**

**June 9,** Sir W. Gowers, **Juvenile Tabes.**

**June 12,** Dr. Tooth, **Multiple Neuritis.**

### The Medical Inspection of School Children.

The following are particulars of the course of lectures and demonstrations which have been arranged for Whit-week in London:

*Tuesday, June* 9:—3 p.m.—Opening meeting in the rooms of the Incorporated Society of Medical Officers of Health. Address by Dr. James Kerr, Chief Medical Officer of the London County Council Education Department.

*Wednesday, June* 10:—11 a.m.—Lecture on "Medical Inspection," by Dr. C. J. Thomas (L.C.C. Education Department). 2 p.m.—Visits to schools. 5.30 p.m.—Lecture, "Anthropometry," Dr. Shrubsall; physical exercises, Dr. Shrubsall.

*Thursday, June* 11:—10.30 a.m.—Demonstration of special cases, Dr. Kerr (L.C.C. Education Offices). 11.30 a.m.—Office routine, ringworm examination, etc., Dr. Thomas (L.C.C. Education Offices); visits to schools. 2.30 p.m.—Lecture, "Eyes and Ears," Dr. Kerr. 3.30 p.m.—Lecture, "School and Home: Infectious Diseases," Dr. H. Meredith Richards. 5.30 p.m.—Lecture, "School Clinics," Dr. Hogarth.

*Friday, June* 12:—10.30 a.m.—Demonstration at London County Council Education Offices by Dr. Kerr; visits to schools.

An exhibition of appliances, outfits, record systems, office requisites, etc., will be open daily from 10 a.m. to 6 p.m. All desirous of attending the lectures are requested to communicate with Mr. William A. Lawton, at the offices of the Incorporated Society of Medical Officers of Health, No. 1 Upper Montague Street, Russell Square, London, W.C.

